# Optimization of the ultrasound-assisted extraction of flavonoids and the antioxidant activity of Ruby S apple peel using the response surface method

**DOI:** 10.1007/s10068-022-01150-8

**Published:** 2022-09-02

**Authors:** NaYeong Park, Sun-Duk Cho, Min-Sun Chang, Gun-Hee Kim

**Affiliations:** 1 Department of Bio-Health Convergence, Duksung Women’s University, Seoul, Republic of Korea; 2 Department of Food and Nutrition, Duksung Women’s University, 33, Samyang-ro 144-gil, Dobong-gu, Seoul, 01369 Republic of Korea; 3 Postharvest Technology Division, National Institute of Horticultural and Herbal Science, RDA, Wanju, Republic of Korea

**Keywords:** Ruby S, Antioxidant, Flavonoid, Response surface method (RSM), Ultrasound-assisted extraction (UAE)

## Abstract

A Box–Behnken Design (BBD) was employed to optimize the extraction of antioxidants from Ruby S apple peel by ultrasound-assisted extraction (UAE). The effect of extraction temperature (20–40 °C), extraction time (15–45 min), and ethanol concentration (50–90%) in water on extraction yield, total phenol content (TPC), total flavonoid content (TFC), and DPPH radical scavenging activity of Ruby S peel extracts (RPEs) were investigated. The optimized extraction conditions that maximized extraction yield, TPC, TFC, and DPPH radical scavenging ability, were temperature 20 °C, extraction time 25.30 min, and ethanol concentration 50%. The validity of designed model was verified, and experimental values obtained under optimum conditions concurred with predicted values. Hyperoside, isoquercitrin, and phloridzin, were among the major flavonoids extracted. Our findings demonstrate the suitability of UAE and RSM for the optimization of Ruby S peel extraction and suggest the potential use of RPEs as bioactive functional materials.

## Introduction

The apple is a perennial woody plant belonging to the *Rosaceae* family and cultivate worldwide, also is one of the representative fruits in Korea that accounted for about 76% of total fruit production in 2020 (Yoon, 2021). As the recent consumption trend that centers on convenience and preferences, the cultivation of recently developed, small-size apples are increasing (Yoon, 2021). Furthermore, it has been reported that these apples are rich in phenolics. Small-to-medium sized apple are not only convenient and easily consumed, but their peels have bioactive effects, which increases their nutritional values. Ruby S (*Malus domestica* Borkh.) is a new apple variety developed in 2014 by the National Institute of Horticultural Research of the Korean Rural Development Administration. It has an average weight of 86 g and excellent storage properties. In addition, studies have reported that Ruby S extracts have antioxidant, anti-inflammatory, gout inhibitory, anti-diabetes, and whitening effects (Lee et al., 2018), which suggests their potential use as a functional material.

Interest in functional materials continues to grow, and many studies have been performed on natural antioxidants (Gulcin, 2020) with the object of targeting reactive oxygen species in vivo. Phenolic compounds are representative natural antioxidants and are known to be present in large amounts in plants (Dai and Mumper, 2010). Flavonoids are found in all plant parts and are characterized by a C6–C3–C6 ring (A, B, and C ring) and 15 carbon atom skeletons. The flavonoid family is composed of several subgroups which include flavonols, flavones, flavanones, chalcones, and isoflavones (Raffa et al., 2017). Apples are rich in these phenolics, and several studies have reported that the phenolic compounds found in apples, which include quercetin, phloridzin, catechin, procyanidin, and rutin are effective at preventing various cancers, degenerative diseases, and free radical-induced aging (Lee et al., 2019).

The extraction method used is important in terms of obtaining these phenolic compounds from plants, and ultrasound-assisted extraction (R) is faster, cheaper, and has better extraction yields than other methods, and is considered a suitable method for extracting phenolics and antioxidants from plants (Park et al., 2020). The extraction parameters must be optimized for different situations, and the response surface method (RSM) is widely used for this purpose in the food industry because it reduces the amount of work required to evaluate interactions between factors and allows complex interactions to be evaluated (Zulkifli et al., 2020).

Many studies have been conducted on the antioxidant components of apple, but no study has addressed the optimal extraction conditions that maximize extract yields for Ruby S. This study is aimed to establish optimal UAE extraction conditions for Ruby S peel that maximize yield, TPC, TFC, and DPPH radical scavenging activity using RSM. After optimization, flavonoids of Ruby S peel extracts were identified by UPLC-ESI-QTOF-MS, and major compounds were quantified by HPLC–DAD.

## Materials and methods

### Sample preparation and chemicals

Ruby S apples were harvested in Andong-si, Gyeongsangbuk-do, Korea in November 2020. Suitable apples were selected for experiments and stored at 4 °C on the day of harvesting. Apples were washed and treated with 1% ascorbic acid and then peeled to separate peel and pulp. The apple peel was freeze-dried (FD8512, Ilshinbiobase, Yangju, Korea) and ground to uniform size using a multiprocessor. Powdered sample was kept in a freezer at − 70 °C for UAE experiments.

The following chemicals were purchased from Sigma-Aldrich (St. Louis, MO, USA): gallic acid, sodium carbonate, Folin–Ciocalteau reagent, naringin, diethylene glycol, sodium hydroxide, sodium acetate and 2,2-diphenyl-1-picrylhydrazyl (DPPH), formic acid, methanesulfonic acid, sodium sulfate, acetonitrile (ACN; HPLC grade), and standards (hyperoside, isoquercitrin, and phloridzin; all HPLC grade). All chemicals used in the experiments were of analytical grade except where mentioned. Ultra-pure water was prepared using the Milli-Q water purification system (Millipore Co., Bedford, MA, USA).

### Ultrasound assisted extraction process

The powdered Ruby S apple peel was extracted to determine the optimal extraction conditions by using an ultrasound assisted extraction (Branson 8510, Branson, USA) at 40 kHz. Powdered apple peel (0.5 g) was added to 10 mL of ethanol (Kim et al., 2019, 2020) and the extracts were obtained in 15-different combinations with 3-levels of extraction temperature (°C), extraction time (min), and ethanol concentration (%) according to experimental design. After the extracts were subsequently centrifuged at 2700 rpm for 15 min and supernatants were collected, they were filtered through Whatman No. 1 filter paper. The filtrate was concentrated using a rotary vacuum evaporator (EYELA Co, Tokyo, Japan) and water bath (EYELA Co, Tokyo, Japan) at their corresponding extraction temperatures, and then freeze-dried. The extracts were stored at -70°C until required for analysis.

### Experimental design

In this study, the experiment used to optimize UAE conditions for extracting antioxidants from Ruby S peel was designed using a BBD. The BBD involved 15 experimental runs to optimize UAE conditions. The following independent extraction variables (${X}_{n}$) were varied (Table 1), namely, extraction temperature (${X}_{1}$: 20, 30 and 40 °C), extraction time (${X}_{2}$: 15, 30 and 45 min), and solvent concentration (${X}_{3}$: 50, 70 and 90%). Extraction yield (${Y}_{1}$), total phenol content (${Y}_{2}$), total flavonoid content (${Y}_{3}$), and DPPH radical scavenging activity (${Y}_{4}$) were designated dependent variables. The ranges of extraction variables were determined through preliminary experiments and literature review. Experiments were conducted randomly.

**Table 1:** Box–Behnken design of extraction parameters for the optimization of yield, TPC, TFC and DPPH radical scavenging activities from Ruby S apple peel extracts obtained by UAE

Run	Independent variables			Dependent variables			
	X_1_	X_2_	X_3_	Y_1_	Y_2_	Y_3_	Y_4_
1	40 (+ 1)	30 (0)	50 (− 1)	37.9 ± 0.20^b^	2.51 ± 0.06^a^	3.80 ± 0.01^d^	70.29 ± 1.62^b^
2	20 (− 1)	30 (0)	90 (+ 1)	31.5 ± 0.17^ k^	1.38 ± 0.03^i^	3.19 ± 0.01^ g^	56.15 ± 2.12^ g^
3	20 (− 1)	30 (0)	50 (− 1)	38.7 ± 0.20^a^	2.49 ± 0.11^a^	4.07 ± 0.02^a^	76.34 ± 0.03^a^
4	40 (+ 1)	30 (0)	90 (+ 1)	34.0 ± 0.20^i^	1.70 ± 0.04^ g^	2.72 ± 0.06^j^	63.49 ± 0.58^e^
5	30 (0)	30 (0)	70 (0)	35.0 ± 0.17^ g^	2.28 ± 0.01^bc^	3.61 ± 0.02^e^	66.37 ± 0.04^d^
6	30 (0)	15 (− 1)	90 (+ 1)	34.5 ± 0.15^ h^	1.50 ± 0.00^ h^	2.93 ± 0.01^i^	61.36 ± 0.17^f^
7	20 (− 1)	45 (+ 1)	70 (0)	35.5 ± 0.10^f^	2.03 ± 0.08^f^	3.84 ± 0.02^c^	63.51 ± 0.38^e^
8	30 (0)	45 (+ 1)	50 (− 1)	37.5 ± 0.20^c^	2.50 ± 0.08^a^	3.83 ± 0.01^ cd^	68.24 ± 0.15^c^
9	30 (0)	30 (0)	70 (0)	35.5 ± 0.17^f^	2.11 ± 0.02^ef^	3.61 ± 0.00^e^	65.97 ± 0.52^d^
10	30 (0)	30 (0)	70 (0)	34.0 ± 0.26^i^	2.12 ± 0.02^def^	3.61 ± 0.01^e^	65.73 ± 0.40^d^
11	30 (0)	45 (+ 1)	90 (+ 1)	33.5 ± 0.17^j^	1.55 ± 0.03^ h^	3.00 ± 0.01^ h^	56.26 ± 0.32^ g^
12	20 (− 1)	15 (− 1)	70 (0)	36.5 ± 0.20^d^	2.17 ± 0.07^de^	3.98 ± 0.01^b^	66.95 ± 0.39^d^
13	30 (0)	15 (− 1)	50 (− 1)	39.0 ± 0.20^a^	2.22 ± 0.01^ cd^	3.95 ± 0.01^b^	71.37 ± 0.26^b^
14	40 (+ 1)	45 (+ 1)	70 (0)	35.0 ± 0.17^ g^	2.36 ± 0.07^b^	3.42 ± 0.02^f^	64.01 ± 0.53^e^
15	40 (+ 1)	15 (− 1)	70 (0)	36.0 ± 0.10^e^	2.20 ± 0.06^cde^	3.44 ± 0.01^f^	66.44 ± 0.33^d^

Data are expressed as mean ± standard deviation (SD) (n = 3)

Different letters in the same columns indicate statistically different results according to Duncan’s multiple range test (*p* < 0.05)

X_1_: extraction temperature (°C); X_2_: extraction time (min); X_3_: ethanol concentration (%); Y_1_: total phenolic content (mg GAE/g DW); Y_1_: total flavonoid content (mg NAE/g DW); Y_3_: DPPH radical scavenging activity (% Inhibition)

Response surface analysis results of relationships between independent and dependent variables were fitted to the following second-order polynomial model [Eq. (1)]:

1 $$ Y = \beta_{0} + \mathop \sum \limits_{i = 1}^{k} \beta_{i} X_{i} + \mathop \sum \limits_{i = 1}^{k} \beta_{ii} X_{i}^{2} + \mathop \sum \limits_{i = 1}^{k - 1} \mathop \sum \limits_{j = i + 1}^{k} \beta_{ij} X_{i} X_{j} $$

where, ${Y}_{1}$, ${Y}_{2}$, ${Y}_{3}$ are dependent variables, ${X}_{1}$, ${X}_{2}$, ${X}_{3}$ are independent variables, ${\beta }_{0}$ is constant, ${\beta }_{i}$, ${\beta }_{ii}$, and ${\beta }_{ij}$ are linear, quadratic, and interaction coefficients, respectively.

Predictions of optimal UAE conditions required to extract antioxidants from Ruby S peel were performed within the range in which extraction yield, total phenol content, total flavonoid content, and DPPH radical scavenging activity values were maximized as determined by RSM. After setting an arbitrary point within the predicted range, optimum values were predicted by substitution into regression equations, and then verification of determined optimal conditions were obtained by comparing predicted and experimental values.

### Extraction yield of sample extracts

Extraction yield of sample was determined by the percentage of the weight of freeze-dried extracts from the total weight of the dried raw samples. The extraction yield was calculated using the Eq. (2).

2 $$ Yield \left( \% \right)\, = \,\frac{Freeze \,dried\, extracts \left( g \right)}{{Dried \,raw\, sample \left( g \right)}}\, \times \,100 $$

### Total phenolic contents

The total phenolic contents (TPC) in extracts were determined by colorimetric analysis using Folin-Ciocalteu reagent, as previously described (Stratil et al., 2006), with several modifications. Extracts 50 μL were reacted with 50 μL of Folin-Ciocalteu’s phenol reagent for 3 min in a 96-well plate, treated with 150 μL of 2% sodium carbonate (w/v) per well, and then incubated for 2 h in a dark room at room temperature. Absorbances were measured using microplate reader (SpectraMax M2, Molecular Devices, USA) at 760 nm. Concentrations were determined using a gallic acid standard calibration plot, and results are expressed as gallic acid equivalents (GAE) in milligrams per gram of dried samples.

### Total flavonoid contents

The total flavonoid contents (TFC) in extracts were determined by spectrophotometric method as previously described (Shi et al., 2019) with some modifications. Extracts (20 μL) were mixed with 200 μL of diethylene glycol and 20 μL of 1 N NaOH in a 96-well plate and then incubated for 1 h at 37 °C. Absorbances were measured using microplate reader at 420 nm, and concentrations were determined using a naringin standard calibration curve. Total flavonoid contents are expressed as naringin equivalents (NAE) in milligram per gram of dried samples.

### DPPH radical scavenging activities

DPPH (1,1-diphenyl-2-picrylhydrazyl) radical scavenging activities were used to evaluate sample antioxidant activities, as previously described (Ramos et al., 2003) with minor modifications. Extracts (50 μL) were mixed with 150 μL of 0.3 mM DPPH dissolved in ethanol and then reacted for 30 min at room temperature. Decreases in absorbance were measured using microplate reader at 515 nm, and DPPH radical scavenging activities were defined as follows Eq. (3):

3 $$ {\text{DPPH}}\,{\text{radical }}\,{\text{scavenging }}\,{\text{activity}}\,{ }\left( {{\text{\% Inhibition}}} \right)\, = \,\left[ {1\, - \,({\text{A}}/{\text{B}})} \right]\, \times \,100 $$

where, A is the absorbance of a sample treated with DPPH radical, and B is the absorbance of a DPPH blank.

### Determination of flavonoids using UPLC-ESI-QTOF-MS and HPLC–DAD

Optimally extracted samples were concentrated on a rotary evaporator and reconstituted with distilled water to 100,000 ppm. Dissolved concentrates were diluted and filtered through Whatman 0.45 μm PVDF filter (Whatman Inc., Piscataway, NJ, USA) to determine phenolic compositions. The analysis was performed as previously described (Kim et al., 2020) with modification.

Phenolics in RPEs were identified by UPLC-ESI-QTOF-MS. LC analysis using a Waters® ACQUITY™ Ultra Performance LC system. A Waters Acquity BEH C18 column (1.7 μm, 2.1 mm × 100 mm) was used with a mobile phase consisting of 0.1% formic acid in water (mobile phase A), and 0.1% formic acid in acetonitrile (mobile phase B) at a flow rate 0.2 mL/min using the following gradient conditions: 5% to 10% B (0–10 min) then 10% to 36% B (10–45 min). Detection was performed at 280 nm. The injection volume was 5 μL, and the column oven temperature was 40 °C. MS analysis was conducted on a Waters SYNAPT G2 system with an electrospray ionization (ESI) source operating in negative ionization mode from 100 to 1000 m*/z*. The MS conditions used were cone voltage 40 V, capillary voltage − 2.5 kV, ion source temperature 120 °C, desolvation gas flow 800 L/h at temperature of 350 °C.

HPLC–DAD was conducted to determine differences in flavonoid contents of RPEs extracted under conditions (extraction temperature of 25 °C, an extraction time of 15 min, and an ethanol concentration of 60%) performed in previous study and optimum conditions. To quantify flavonoids in RPEs, HPLC (Thermo Scientific™ UltiMate™ 3000 UHPLC) coupled with UV–Vis/DAD was used with Alltima (Alltech Associates, Inc. Deerfield, IL) C18 analytical column (5 μm, 250 mm × 4.6 mm). Gradient elution was conducted as described above for LC–MS at a flow rate of 0.8 mL/min, an injection volume of 20 μL, at a column temperature of 30 °C using a detection wavelength of 280 nm. Hyperoside, isoquercitrin, and phloridzin, identified as major compounds by LC–MS were analyzed using linear calibration curves of commercial standards: hyperoside (y = 0.6311x − 0.3388, R^2^ = 0.9986), isoquercitrin (y = 0.9702x + 0.1977, R^2^ = 0.9990), and phloridzin (y = 0.7877x + 12.923, R^2^ = 0.9989).

### Statistical analysis

All experiments were carried out in triplicate, and results are expressed as mean ± standard deviation (SD). Statistical analysis was performed using one-way analysis variance (ANOVA) and SPSS ver. 20.0 (SPSS Inc., IL, USA). The significances of differences between means were determined using Duncan’s multiple range test, and statistical significance was accepted for *p* < 0.05. Minitab 19 (Minitab Inc., PA, USA) was used to generate surface plots for optimization experiments.

## Results and discussions

In UAE process, extraction temperature, time, and solvent type significantly influence extraction yield, and also the release of phenolic compounds from solid matrix and the antioxidant activities of extracts (Chemat et al., 2017). Generally, heating process enhances the solubility of the compounds and the diffusion coefficient of solvent, however, some flavonoids such as procyanidins, which is abundant in apples, could be degraded by high temperatures above 50 °C (Escribano-Bailón and Santos-Buelga, 2004). In preliminary experiments for study, TPC results showed no significant difference between 4 °C and room temperature extraction, respectively 1.47 and 1.46 mg GAE/g DW, while the TPC was lowered at 50 °C extraction. Generally, a mixture of solvents and water are more efficient than mono-solvent in phenolic extraction. As a result of preliminary experiments, TPC was 1.94-fold higher in 60% ethanol extraction than distilled water extraction, and 20% and 40% ethanol extraction showed more than 20% lower TPC and TFC than 60% ethanol extraction. Considering the results of the preliminary experiments and the literature review, extraction parameters and response variables were set described below, then 15-run experiments were performed according to the Box-Behnken design model to identify optimal conditions for extracting antioxidants from Ruby S apple peel using UAE. Table 1 shows the mean values of extraction yield, TPC, TFC, and DPPH radical scavenging activity of RPEs obtained at different extraction temperatures, extraction times, and ethanol concentrations.

### Effect of UAE factors on extraction yield

As shown in Table 1, the extraction yield of RPEs obtained under various experimental conditions varied significantly (*p* < 0.05) from 31.5 ± 0.17% (run 2) to 39.0 ± 0.20% (run 13). The maximum value of the extraction yield was obtained at an extraction temperature of 30 °C, an extraction time of 15 min, and an ethanol concentration of 50% (abbreviated to 30 °C/15 min/50% EtOH hereafter). On the other hand, the minimum was observed at 20 °C/30 min/90% EtOH, which was 7.5% lower than the maximum. This finding exceeded that obtained by another study on optimizing extraction method (Nakamura et al., 2019). They found that extraction yield of plant extracts using ultrasonic extraction with on 0–100% ethanol was ranged 3.82 to 27.62% and was decreased to 3.82% on 100% ethanol extraction.

The predicted model for extraction yield could be described in terms of coded factors using the following regression equation:

$$ \begin{aligned} Y_{1} & = 64.80 - 0.375X_{1} - 0.269X_{2} - 0.445X_{3} + 0.00158X_{1}^{ 2} - 0.0337X_{2}^{ 2} + 0.00133X_{3}^{ 2} \\ & \quad + 0.00000X_{1} X_{2} + 0.00413X_{1} X_{3} + 0.00042X_{2} X_{3} \\ \end{aligned} $$

**Fig. 1:**
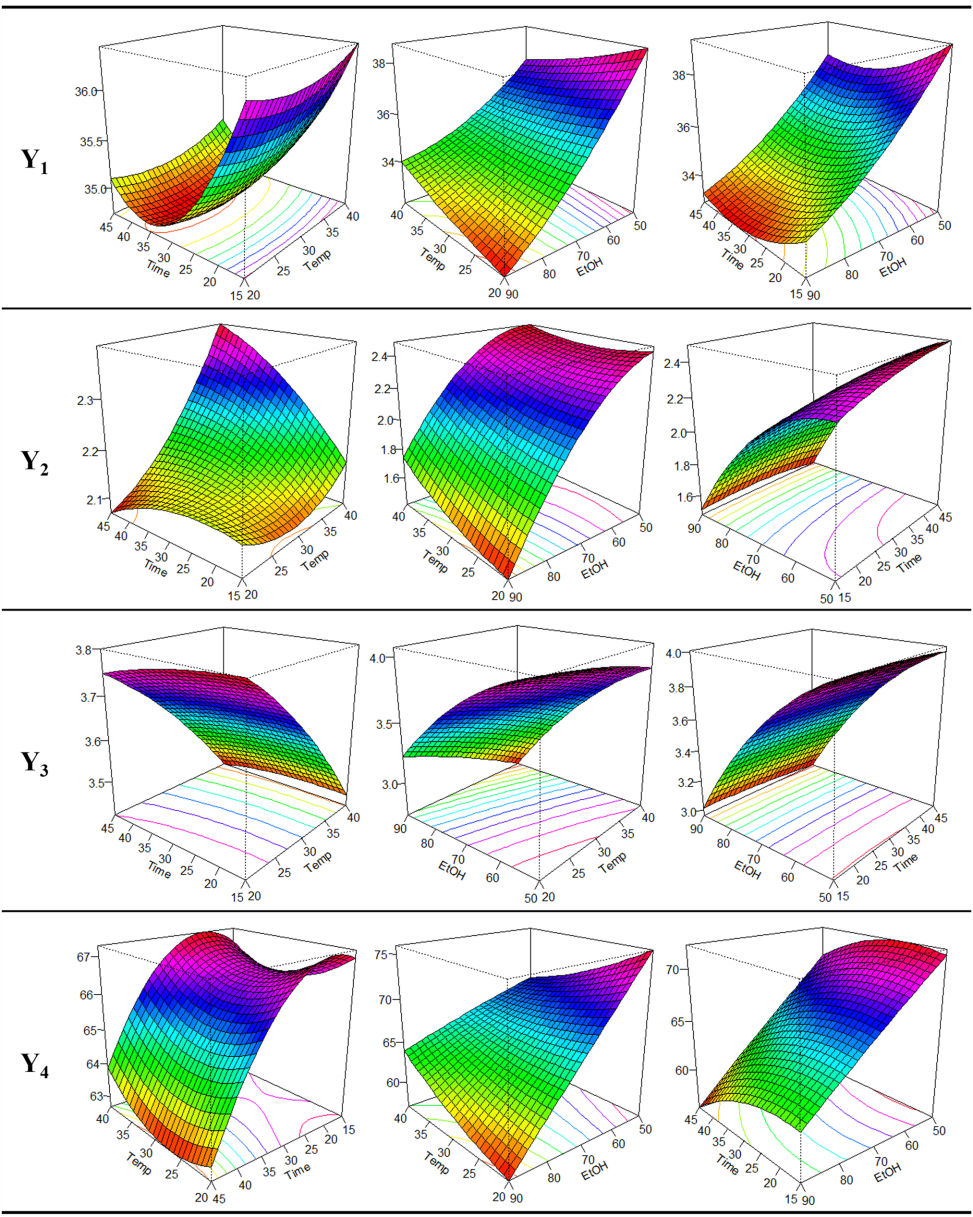
Response surface plots and 2-dimensional contour lines for the effects of the extraction temperature (°C), extraction time (min), and ethanol concentration (%) on the extraction yield (%, Y_1_), TPC (mg GAE/g DW, Y_2_), TFC (mg NAE/g DW, Y_3_), and DPPH radical scavenging activity (% Inhibition, Y_4_) of RPEs

The 3-dimensional response surfaces and 2-dimensional contours obtained by the prediction model are shown in Fig. 1. In the response surface graphs, the fixed values were 30 °C/30 min/70% EtOH. As a result of the response surface analysis, the predicted stationary point was the maximum point, and the maximum value was predicted to be 40.16% at the extraction temperature of 20 °C, extraction time of 15 min, and ethanol concentration of 50%. The higher the extraction temperature and the shorter the extraction time, the higher the extraction yield of RPEs, but it was not statistically significant. Generally, an increase of temperature leads to higher extraction yield, which concurs with a previous study of optimizing UAE conditions for extraction of hazelnut oil (Geow et al., 2018). Meanwhile, extraction yield was significantly higher when the ethanol concentration was reduced (*p* < 0.001). Therefore, ethanol concentration was, the only factor that had a linear effect on extraction yield in the response surface model, was also predicted to be the most significant factor.

### Effect of UAE factors on TPC

The range for TPC of RPEs obtained under different experimental UAE conditions significantly ranged from 1.38 ± 0.11 mg GAE/g DW (run 2) to 2.51 ± 0.06 mg GAE/g DW (run 1) as shown in Table 1 (*p* < 0.05). Maximum TPC was recorded at 40 °C/30 min/50% EtOH and the minimum was observed at 20 °C/30 min/90% EtOH, which was significantly 1.8-times lower than the maximum (*p* < 0.05). This finding showed the similar results as previous study (Lee et al., 2019) indicating decreased TPC at 90% ethanol extraction compared to using 50% ethanol when ‘Picnic’ apple were extracted with various concentration of ethanol.

The predicted model for TPC could be described in terms of coded factors using the following regression equation:

$$ \begin{aligned} Y_{2} & = 2.124 - 0.0629X_{1} + 0.0100X_{2} + 0.0413X_{3} + 0.000507X_{1}^{ 2} - 0.000137X_{2}^{ 2} \\ & \quad - 0.000494X_{3}^{ 2} + 0.000501X_{1} X_{2} + 0.000377X_{1} X_{3} - 0.000197X_{2} X_{3} \\ \end{aligned} $$

The 3-dimensional response surfaces and 2-dimensional contours obtained using the predicted model are shown in Fig. 1. In the response surface graphs, the fixed values were 30 °C/30 min/70% EtOH. The predicted stationary point according to the response surface analysis was the saddle point, and the maximum value was predicted to be 2.63 mg GAE/g DW at the extraction temperature of 40 °C, extraction time of 45 min, and ethanol concentration of 50%. As the extraction temperature and time increased and ethanol concentration decreased, TPC in RPEs increased, especially for temperature (*p* < 0.05) and ethanol concentration (*p* < 0.001). On the other hand, only quadratic parameter ${(X}_{3}^{ 2})$ of ethanol concentration had a significant effect at the *p* < 0.01 level on TPC. As a result, ethanol concentration was found to be the most significant factor, due to increased phenolic solubility (Prgomet et al., 2019). Extraction temperature also had a significant effect on TPC.

### Effect of UAE factors on TFC

The means of TFC of RPEs obtained under the various experimental UAE conditions are shown in Table 1. Values were significantly varied (*p* < 0.05) from 2.72 ± 0.06 mg NAE/g DW (run 4) to 4.07 ± 0.02 mg NAE/g DW (run 3). The maximum and minimum TFC was observed at 20 °C/30 min/50% EtOH and 40 °C/30 min/90% EtOH, respectively, and minimum TFC value was 1.5-fold lower than maximum.

The RPEs predicted model for total flavonoid content can be described in terms of coded factors by the following regression equation:

$$ \begin{aligned} Y_{3} & = 3.085 + 0.02702X_{1} + 0.00217X_{2} + 0.03645X_{3} - 0.000298X_{1}^{ 2} - 0.000053X_{2}^{ 2} \\ & \quad - 0.000350X_{3}^{ 2} + 0.000042X_{1} X_{2} - 0.000394X_{1} X_{3} - 0.000022X_{2} X_{3} \\ \end{aligned} $$

The 3-dimensional response surfaces and 2-dimensional contours designed by the predicted model are shown in Fig. 1. In the response surface graphs, the fixed values were 30 °C/30 min/70% EtOH. The predicted stationary point according to the response surface analysis was the saddle point, and the maximum value was predicted to be 4.08 mg NAE/g DW at the extraction temperature of 20 °C, extraction time of 18.03 min, and ethanol concentration of 50%. A proportional inverse tendency was observed between all extraction parameters and TFC, and this was significant at the *p* < 0.001 level for temperature and ethanol concentration. Meanwhile, all the quadratic terms of extraction parameters $${(X}_{n}^{ 2})$$, excluding extraction time, were significant (*p* < 0.01), which indicated that extraction temperature $${(X}_{2}^{ 2})$$ and ethanol concentration $${(X}_{3}^{ 2})$$ influenced on total flavonoid content. Moreover, the interaction between extraction temperature and ethanol concentration ($${X}_{1}{X}_{3})$$ was significant at the *p* < 0.001 level. Therefore, all parameters influenced total flavonoid content, but extraction temperature and ethanol concentration were the major factors. Furthermore, these results agree with previously reported results (Alberti et al., 2014) that high temperatures degrade flavonoids in apples.

### Effect of UAE factors on DPPH radical scavenging activity

The range for DPPH radical scavenging activity of RPEs obtained using the various experimental UAE conditions significantly ranged from 56.15 ± 2.12% (run 2) to 76.34 ± 0.03% (run 3) as shown in Table 1 (*p* < 0.05). The highest DPPH radical scavenging activity and TFC were observed at 20 °C/30 min/50% EtOH. On the other hand, the lowest DPPH radical scavenging activity was obtained at 20 °C/30 min/90% EtOH, as was observed minimum for extraction yield and TPC, 20.19% lower compared to the maximum. This finding indicating that DPPH radical scavenging activity has correlation with not only extraction conditions, but also extraction yield, TPC, and TFC. In ultrasonic extraction, particle size, solvent to solid ratio, solvent type, ethanol concentration, sonication amplitude and extraction time affects the extraction yield, antioxidant effects, TPC, and TFC. In addition, extraction yield has a significant correlation with TPC, TFC, and antioxidant effect (Lim et al., 2019).

The predicted model for DPPH radical scavenging activity can be described in terms of coded factors by the following regression equation:

$$ \begin{aligned} Y_{4} & = 121.9 - 1.646X_{1} + 0.353X_{2} - 0.693X_{3} + 0.00731X_{1}^{ 2} - 0.00678X_{2}^{ 2} - 0.00047X_{3}^{ 2} \\ & \quad + 0.00170X_{1} X_{2} + 0.01674X_{1} X_{3} - 0.00164X_{2} X_{3} \\ \end{aligned} $$

The 3-dimensional response surfaces and 2-dimensional contours obtained using the predicted model are shown in Fig. 1. In the response surface graphs, the fixed values were 30 °C/30 min/70% EtOH. As a result of the response surface analysis, the predicted stationary point was the saddle point, and the maximum value was predicted to be 76.26% at the extraction temperature of 20 °C, extraction time of 22.58 min, and ethanol concentration of 50%. The effects of extraction time and ethanol concentration on DPPH radical scavenging activity tended to decrease, and ethanol concentration decreased most significantly (*p* < 0.001). Only the quadratic term of extraction time ${(X}_{2}^{ 2})$ was significant, but the interaction between extraction temperature and ethanol concentration ${(X}_{1}{X}_{3})$ also had a significant effect on DPPH radical scavenging activity. Therefore, all parameters affected DPPH radical scavenging activity, and ethanol concentration variable had the strongest effect which agreed with a previous report (Prgomet et al., 2019) that affect antiradical power of the extracts.

### Model fitting and analysis of variance (ANOVA)

Analysis of variance (ANOVA) and multiple regression analysis were conducted (Table 2). The obtained models showed highly significant probability values. The models for TPC, TFC, and DPPH were highly significant at *p* < 0.001 level, and model for yield was significant at *p* < 0.01 level. The regression coefficient of yield was significant only by linear regression of ${X}_{3}$, while other responses were significant by two or more source in linear, quadratic, and interaction regressions. The linear and quadratic regression coefficients of ${X}_{1}$, ${X}_{3}$ and ${X}_{3}^{ 2}$ for TPC had significantly low *p*-values. A similar trend was observed in previous study (Alberti et al., 2014) that quadratic regression coefficient of methanol concentration was significant. The *p*-values of linear and interactive regression of ${X}_{2}$, ${X}_{3}$ and ${X}_{1}{X}_{3}$ were also significantly low for DPPH radical scavenging activity. Unlike other response values, regression coefficients for TFC were significant at the *p* < 0.001 level in all linear regressions (${X}_{1}$, ${X}_{2}$ and ${X}_{3}$), quadratic regression of ${X}_{1}^{ 2}$ and ${X}_{3}^{ 2}$, and interaction regression of ${X}_{1}{X}_{3}$. These findings indicated that extraction yield, TPC, TFC, and DPPH are affected by a single or interaction of temperature, time, and solvent concentration, and this trend was agreed with other studies on UAE condition optimization (Alberti et al., 2014; Mohamed Ahmed et al., 2020).

**Table 2:** Analysis of variance (ANOVA) and fitness of the model regression equation

Source	Yield (%)		TPC (mg GAE/g DW)		TFC (mg NAE/g DW)		DPPH (% Inhibition)	
	SS	*p*-value	SS	*p*-value	SS	*p*-value	SS	*p*-value
Model	56.38	0.009**	1.91	0.001***	2.35	0.000***	382.38	0.000***
Linear	50.61	0.001**	1.69	0.000***	2.25	0.000***	324.96	0.000***
${X}_{1}$	0.06	0.760	0.06	0.032*	0.24	0.000***	0.21	0.645
${X}_{2}$	2.53	0.093	0.02	0.204	0.00	0.009**	24.88	0.003**
${X}_{3}$	48.02	0.000***	1.61	0.000***	2.01	0.000***	299.87	0.000***
Quadratic	2.98	0.285	0.16	0.028*	0.07	0.000***	11.34	0.071
${X}_{1}^{ 2}$	0.02	0.709	0.02	0.308	0.00	0.008**	2.72	0.188
${X}_{2}^{ 2}$	1.91	0.116	0.00	0.522	0.00	0.149	8.49	0.025*
${X}_{3}^{ 2}$	1.05	0.240	0.14	0.007**	0.07	0.000***	0.13	0.712
Interaction	2.79	0.307	0.06	0.158	0.03	0.000***	46.09	0.004**
${X}_{1}{X}_{2}$	0.00	1.000	0.02	0.141	0.00	0.388	0.26	0.604
${X}_{1}{X}_{3}$	2.72	0.085	0.02	0.139	0.02	0.000***	44.85	0.001***
${X}_{2}{X}_{3}$	0.06	0.758	0.01	0.227	0.00	0.364	0.97	0.334
Lack of fit	1.79	0.529#	0.02	0.618#	0.00	0.320#	4.05	0.072#
R^2^	0.9502		0.9811		0.9996		0.9890	
Adjusted R^2^	0.8606		0.9469		0.9989		0.9692	
PRESS	31.23		0.35		0.01		65.25	

*SS* sum of square; *PRESS* prediction error sum of squares; *TPC* total phenolic content; *TFC* total flavonoid content; *DPPH* DPPH radical scavenging activity; *GAE* gallic acid equivalents; *NAE* naringin equivalents

*p < 0.05, **p < 0.01, ***p < 0.001 indicates significance; ^#^*p* of lack of fit indicates not significance (low standard errors)

The results of the analysis performed to assess the fitness of models are also summarized in Table 2. The regression coefficients of determination (R^2^) of models to evaluate the quality of models were 0.9502, 0.9811, 0.9996, and 0.9890 for yield, TPC, TFC, and DPPH, respectively. These results indicate that only 4.98%, 1.89%, 0.04%, and 1.1% of the total variabilities of responses for yield, TPC, TFC, and DPPH could not be explained by the model. The adjusted R^2^ values of models were 0.8606 for yield, 0.9469 for TPC, 0.9989 for TFC, and 0.9692 for DPPH. These models showed the better results than earlier study (Alberti et al., 2014) of optimizing UAE of phenolic compounds from apples using methanol that explained ${R}_{Adj}^{2}$ of TPC model for 0.80, TFC for 0.82, and DPPH radical scavenging activity for 0.94. The lack-of-fit test (*p* > 0.05) indicated that the suitability of each model accurately predicted variations (Jibril et al., 2019). All models of responses were suitable, *p*-values obtained for the lack-of-fit test were 0.529 for yield, 0.618 for TPC, 0.320 for TFC, and 0.072 for DPPH radical scavenging activity. The prediction error sum of squares (PRESS) provides a measure of the deviation between fitted and observed values. In general, a smaller PRESS value indicates better model predictive ability (Kumar et al., 2019). The PRESS values of models were 31.23 for yield, 0.35 for TPC, 0.01 for TFC, and 65.25 for DPPH, which showed models of yield, TPC, TFC, and DPPH were suitable.

### Optimization and verification of UAE conditions

Optimization was performed to determine extraction conditions that simultaneously maximize UAE extraction yield, total phenolic content, total flavonoid content, and DPPH radical scavenging activity in RPEs. The polynomial models established in this study were utilized to obtain optimal UAE conditions, and to predict values that maximized simultaneously responses of the above four variables.

The optimal conditions for simultaneously maximizing all four responses (extraction yield, TPC, TFC, and DPPH radical scavenging activity) were 20 °C/25.3 min/50% EtOH. Under these extraction conditions, the fit was 39.00% for extraction yield, 2.44 mg GAE/g DW for TPC, 4.07 mg NAE/g DW for TFC, and 76.20% for DPPH radical scavenging activity. The 95% confidence intervals (CI) of predicted values were 37.25–40.76 (%) for extraction yield, 2.25–2.64 (mg GAE/g DW) for TPC, 4.03–4.10 (mg NAE/g DW) for TFC, and 74.10–78.31 (% Inhibition) for DPPH radical scavenging activity. In the previous study (Lee et al., 2018) of extracting Ruby S apple peels by a conventional extraction method, low temperature extraction for 24 h, the TPC was 8.76 mg GAE/g, which was superior to this study, but DPPH radical scavenging activity value was superior in this study. The difference in the TPC is attributed to the fact that even if the same variety is used, the polyphenol contained in the fruit varies depending on various factors such as the maturity, harvest time, color, and processing method (Rice-Evans et al., 1997).

Individual and composite desirability is an indicator that ranges from 0 (undesirable response) to 1 (desirable response) and is used to assess how well a combination of variables satisfies goals (Maran et al., 2015). This indicator is used to assess how well settings optimize single or a series of responses. In the present study, the individual desirability of all responses was almost 1 (yield: 1.00, TPC: 0.94, TFC: 1.00, DPPH: 0.99), and the composite desirability was 0.983; a near ideal result.

Experiments to compare the mean values of experimental and predicted results to verify the suitability of the model were performed in triplicate under optimized conditions. The experimental results obtained under optimal conditions were 38.17 ± 1.04% for extraction yield, 2.45 ± 0.01 mg GAE/g DW for TPC, 4.09 ± 0.05 mg NAE/g DW for TFC, and 77.52 ± 2.23% for DPPH radical scavenging activity, and these results were well-matched to predicted results and were valid within 95% CI of predicted values, which confirmed the suitability of devised model. In addition, the obtained optimal conditions were different with earlier study (Alberti et al., 2014) on optimization of extracting phenolic compounds from apple that using methanol and acetone for solvent, but absolute errors between predicted value and observed value were similar or better in this study.

### Identification and quantification of flavonoids in RPEs

**Fig. 2:**
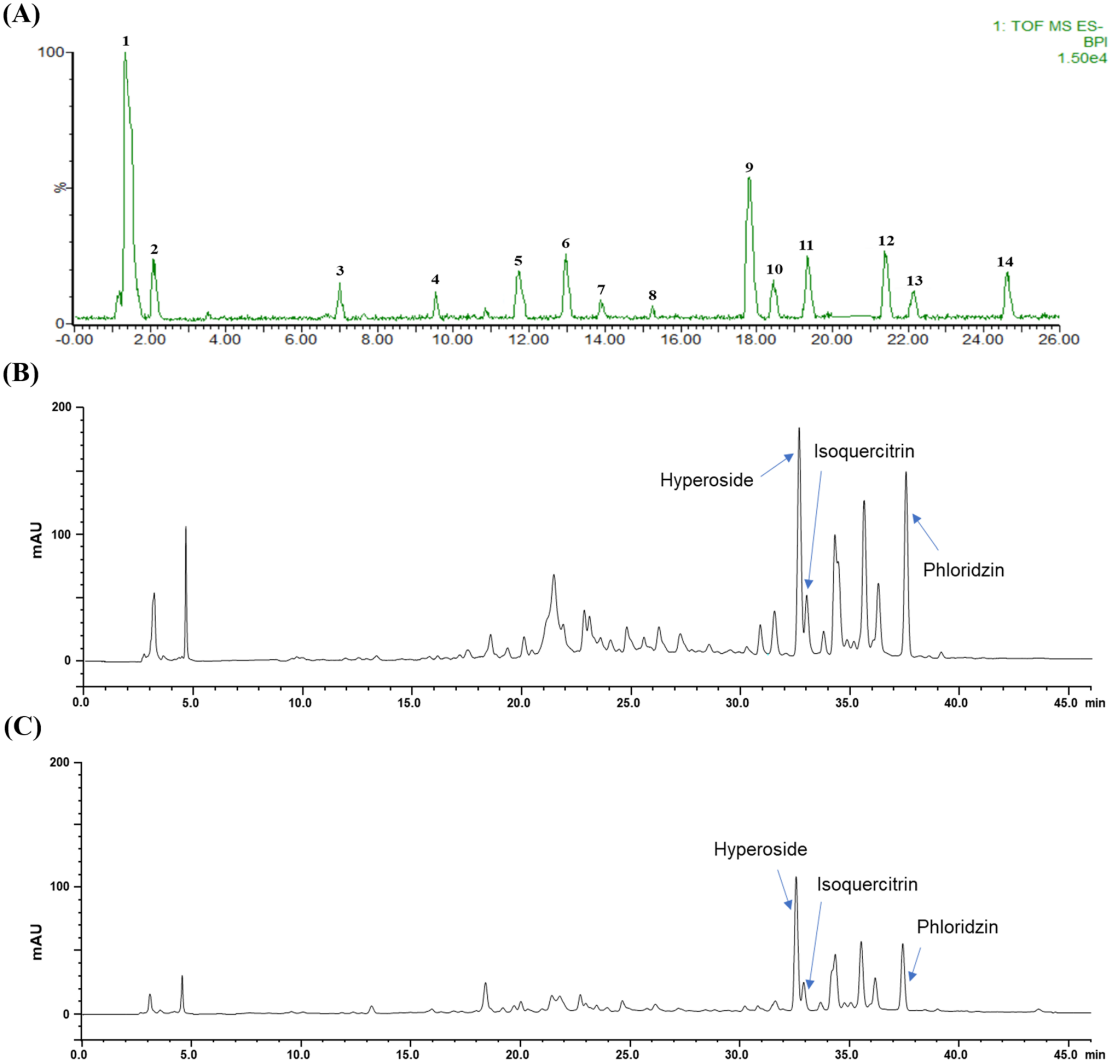
LC–MS and HPLC–DAD chromatograms at 280 nm of flavonoids obtained from RPEs. (**A**) is LC–MS chromatogram and the identities of the 14 peaks are given in Table 3, (**B**) and (**C**) are HPLC–DAD chromatogram. (**B**) is of RPEs obtained using optimum extraction conditions and (**C**) is of P-REPs obtained using previous extraction conditions

The flavonoids detected in the RPEs are shown in a chromatogram (Fig. 2A) and are listed in Table 3. The identification of phenolic compounds was conducted by comparing retention times, UV spectra, and MS data, and theoretical fragmentation data reported in the literature. Fourteen peaks were detected and included 4 flavonols, 2 flavan-3-ols, 2 dihydrochalcones, 1 flavones, and five unknowns (peaks 1, 2, 4 and 5).

**Table 3:** Profile of flavonoids in Ruby S peel as detected by UPLC-ESI-QTOF-MS

Peak	Rt (min)	[M-H]^−^ (*m/z*)	Molecular weight	Molecular formula	Compound name	Compound class
1	1.33	341.1515	–	–	Unknown	–
2	2.08	147.0940	–	–	Unknown	–
3	6.99	457.1934	458.37	C_22_H_18_O_11_	(Epi)gallocatechin gallate	Flavan-3-ols
4	9.54	411.2180	–	–	Unknown	–
5	11.74	427.2080	–	–	Unknown	–
6	12.96	563.2297	564.49	C_26_H_28_O_14_	Apigenin-7-(2-*O*-apiosylglucoside)	Flavones
7	13.89	483.2245	–	–	Unknown	–
8	15.26	441.2241	442.37	C_22_H_18_O_10_	(Epi)catechin gallate	Flavan-3-ols
9^a^	17.82	463.1078	464.38	C_21_H_20_O_12_	Quercetin-3-*O*-galactoside (hyperoside)	Flavonols
10^a^	18.44	463.1071	464.38	C_21_H_20_O_12_	Quercetin-3-*O*-glucoside (Isoquercitrin)	Flavonols
11	19.34	433.1034	434.35	C_20_H_18_O_11_	Quercetin pentoside	Flavonols
12	21.38	447.1161	448.38	C_21_H_20_O_11_	Kaempferol hexoside	Flavonols
13	22.16	567.1691	568.52	C_26_H_32_O_14_	Phloretin pentosylhexoside	Dihydro-chalcones
14^a^	24.62	435.1548	436.41	C_21_H_24_O_10_	Phloridzin	Dihydro-chalcones

^a^Compound identities confirmed with commercial standards

The four flavonols were detected at retention times from 17.82 to 21.38 min, that is, quercetin-3-*O*-galactoside (hyperoside) at *m/z* 463.1078 (17.82 min), quercetin-3-*O*-glucoside (isoquercitrin) at *m/z* 463.1071 (18.44 min), quercetin pentoside at *m/z* 433.1034 (19.34 min), and kaempferol hexoside at *m/z* 447.1161 (21.38 min). Hyperoside and isoquercitrin are isomerized form, and are indistinguishable only by MS^1^, and thus, were identified by referring to literature MS^2^ data (Kim et al., 2019) obtained using the identical conditions and by comparing retention times with authentic standards. Previous studies reported that hyperoside and isoquercitrin are predominant in apple peel, including Ruby S peel. In agreement with earlier studies (Raffa et al., 2017; Stefova et al., 2019), which identified phenolic compounds in apple peel, pulp, and leaves, quercetin pentoside and kaempferol hexoside were detected in Ruby S apple peel. Quercetin pentoside is believed to be avicularin (quercetin 3-α-l-arabinofuranoside) or guajaverin (quercetin-3-*O*-arabino-pyranoside) and is known to be present in apples (Sánchez-Rabaneda et al., 2004). All detected flavonols were aglycones with pentose or hexose bound to quercetin or kaempferol. Flavonol glycosides were the most detected of the four subclasses, which agreed with a previous report that flavonol glycosides are predominant in apples (Raudone et al., 2017). (Epi) gallocatechin gallate (*m/z* 457.1934) and (epi) catechin gallate (*m/z* 441.2241) were the most detected flavan-3-ols at retention times of at 6.99 and 15.26 min, respectively (Zhang et al., 2014). Epigallocatechin gallate and epicatechin gallate are isomeric with gallocatechin gallate and catechin gallate, respectively, and cannot be positively identified by conventional LC–MS/MS, but are detectable by using MS with hydrogen/deuterium exchange (Susanti et al., 2015), and thus, were considered these identifications as tentative. Furthermore, our identification of epicatechin and catechin derivatives in apple peel agreed with previous studies that total catechins are abundant in apple peel and that they are the major phenolic compounds in apple peel (Kim et al., 2019). Only one flavone derivative was found at a retention time of 12.96 min, which corresponded with a deprotonated molecular ion at *m/z* 563.2297 assigned to apigenin-7-(2-*O*-apiosyl-glucoside) (Tang et al., 2020). Apigenin and apigenin-7-glucoside have been reported in apple leaves and the detected apigenin-7-(2-*O*-apiosylglucoside) (apiin) is a diglycoside of the flavone apigenin (Petkovska et al., 2017; Stefova et al., 2019). Two compounds were identified as phloretin derivatives. Peak 13 (phloretin pentosylhexoside) was detected at a retention time of 22.16 min and had a deprotonated molecular ion at *m/z* 567.1691. It was tentatively identified as phloretin-2-xylosylglucoside, which has been reported in apples (He et al., 2022; Montero et al., 2013). Peak 14 had a retention time of 24.62 min and [M–H]^−^ *m/z* peak at 435.1548, which was confirmed to be phloridzin using authentic standard. Both compounds have been reported to be present in apple peel, flesh, and leaves by analytic studies on phenolic compounds in apples (Kim et al., 2019; Montero et al., 2013; Stefova et al., 2019).

HPLC–DAD at 280 nm was performed to quantify levels of hyperoside, isoquercitrin, and phloridzin levels, which were identified as major components by UPLC-QTOF-MS. Comparisons of major flavonoid contents in RPEs extracted under optimized condition and previous study condition (P-RPEs) extracted at 25 °C/15 min/60% EtOH were conducted to evaluate the efficiency of extracting major components. The UV–Vis chromatograms are shown in Fig. 2B and C. Three of major flavonoids were detected at relatively high concentrations in RPEs, and HPLC–DAD peaks concurred with LC–MS results (Fig. 2A). These results are in line with the tendency for TFC to be higher under optimum condition (4.09 mg NAE/g DW) than previous study (3.83 mg NAE/g DW) extracted at described above conditions. For RPEs extracted under optimized conditions, hyperoside was found to be 1.8-fold (121.50 ± 3.17 μg/g) and isoquercitrin to be 2.8-fold (22.27 ± 2.06 μg/g) significantly (*p* < 0.01) higher than P-RPEs (hyperoside: 67.62 ± 3.17 μg/g, isoquercitrin: 7.84 ± 2.06 μg/g). In particular, a large amount of phloridzin was extracted from optimized conditions, that is 10-times (52.27 ± 2.81 μg/g) significantly (*p* < 0.001) higher than P-RPEs (5.24 ± 3.72 μg/g), and this result concurs with (Choi and Chung, 2019) at green apple extracted with 50% ethanol showed the highest contents of polyphenol and phloridzin. These results indicate that optimal UAE condition is effective to extract antioxidants, especially flavonoids, and it could be served as a useful background for the large-scale optimization of extraction methods for food industrial.
